# Survival outcomes of minimally invasive versus open radical hysterectomy in patients with early-stage IB1 to IIA2 cervical cancer: A single-center retrospective study

**DOI:** 10.1097/MD.0000000000033702

**Published:** 2023-04-28

**Authors:** Hwa Yeon Choi, Jung-Woo Park

**Affiliations:** a Department of Obstetrics and Gynecology, Dong-A University College of Medicine, Busan, Republic of Korea.

**Keywords:** cervical cancer, disease-free survival, minimally invasive surgery, overall survival, radical hysterectomy

## Abstract

This study aimed to investigate the survival outcomes and prognostic factors associated with the surgical approach in patients with early-stage cervical cancer. We retrospectively analyzed 245 patients with stage IB1 to IIA2 cervical cancer who underwent radical hysterectomy with pelvic lymphadenectomy between 2004 and 2019 at Dong-A University Hospital. A total of 59 patients underwent minimally invasive surgery (MIS), and 186 patients underwent open surgery. There were no significant differences between the 2 groups, except for stromal invasion (*P* < .001), lymphovascular invasion (*P* = .001), and requirement for adjuvant therapy (*P* < .001). There were no significant differences in disease-free survival (DFS) and overall survival (OS) based on the surgical approach. However, multivariate analyses showed MIS was an independent poor prognostic factor of DFS (adjusted hazard ratio [HR]: 230; 95% confidence interval [CI]: 086–0.614, *P* = .003) and OS (adjusted HR: 135; 95% CI: 041–0.451, *P* = .001). Adjuvant therapy was a poor prognostic factor for DFS (adjusted HR: 6.546; 95% CI: 1.384–30.952; *P* = .018), and deep stromal invasion was a poor prognostic factor for OS (adjusted HR: 8.715; 95% CI: 1.636–46.429; *P* = .01). MIS may be an independent poor prognostic factor for DFS and OS in patients who undergo radical hysterectomy for early-stage cervical cancer.

## 1. Introduction

Radical hysterectomy with pelvic lymph node dissection is the mainstay treatment for early-stage cervical cancer, even in patients with stages IB3 and IIA2 according to the 2018 Federation International of Gynecology and Obstetrics (FIGO) staging.^[1–3]^ Minimally invasive radical hysterectomy has emerged as a novel treatment approach with associated advantages of lesser estimated blood loss, shorter hospital stays, and comparable survival outcomes relative to traditional open abdominal radical hysterectomy.^[4,5]^ However, the Laparoscopic Approach to Cervical Cancer (LACC) trial showed that minimally invasive radical hysterectomy was inferior to open abdominal radical hysterectomy in terms of disease-free survival (DFS) and overall survival (OS).^[6]^ After the LACC trial, the revised National Comprehensive Cancer Network guideline recommended the open approach as the standard radical hysterectomy approach.^[1]^ Thus, there is uncertainty regarding the selection between minimally invasive and open surgical approaches to radical hysterectomy. Therefore, in this study, we aimed to investigate the survival outcomes associated with surgical approaches in patients with early-stage cervical cancer based on the 2018 FIGO staging at a single center. Furthermore, we aimed to evaluate prognostic factors affecting the survival outcomes.

## 2. Materials and methods

### 2.1. Study population

We screened patients registered at our gynecologic cancer center and identified those with early-stage cervical cancer (clinical FIGO stage IB1–IIA2 disease) who underwent primary surgical treatment at the Dong-A University Hospital between April 2004 and April 2019. We retrospectively reviewed the patients’ demographic, clinical, surgical outcomes, and pathological data. The changed stage was included in the data by applying the revised FIGO staging system of 2018.

The inclusion criteria were as follows: pathologically confirmed cervical cancer, clinically diagnosed FIGO stage IB to IIA, and history of radical hysterectomy type C and pelvic lymphadenectomy. The following patients were excluded from the present study: those who received neoadjuvant chemotherapy before surgery, those with a history of radiation therapy, those with incidental findings after simple hysterectomy, and those with missing or insufficient clinical or pathological data.

### 2.2. Data collection and definitions

We surveyed medical records and pathologic and radiologic examinations, and collected information regarding the clinicopathological findings (age, tumor size, FIGO stage, types of histology, risk factors of pathologic reports, surgeries on pelvic lymph nodes, and methods of adjuvant treatment). The tumor size was determined as the maximal diameter by inspection. In cases with no clear clinical information, the tumor size was measured using magnetic resonance imaging and/or computed tomography. All stages were reassigned using the new FIGO classification of 2018, as patients were registered before 2018. We obtained pathological risk factors, such as depth of invasion (divided into thirds), lymphovascular space invasion, involvement of pelvic lymph nodes, parametrial involvement, and invasion of the resected margin.

All patients underwent regular physical and radiographic examinations. For imaging studies during surveillance, chest radiography, magnetic resonance imaging, or computed tomography scans were performed every 6 months for the first 3 years and every 12 months thereafter, if indicated based on symptoms or examination findings suggestive of recurrence.

DFS was defined as the time interval from surgery to the first evidence of recurrence and date of disease progression. OS was defined as the time interval from the date of initial diagnosis to the date of cancer-related death or last follow-up examination.

### 2.3. Statistical analyses

We evaluated the differences in clinicopathological findings between patients who underwent minimally invasive surgery (MIS) and those who underwent open surgery. We used the mean (standard deviation) to compare continuous variables. Student *t* test was used to compare mean values. Categorical variables are presented as frequencies (percentages). Pearson chi-square test and Fisher exact test were used to analyze the distribution of characteristics according to the surgical approach. Survival outcomes were calculated using Kaplan–Meier curve analysis and compared using the log-rank test. Cox regression analysis was performed to identify the prognostic factors for survival outcomes using backward elimination of variables based on the Wald test. Variables included in the multivariate Cox regression analysis were those found to be significant (*P* < .10) in the univariate Cox regression analysis. We defined the threshold for statistical significance as *P* < .05. All statistical analyses were performed using SPSS software (version 22.0; IBM SPSS Statistics, IBM Corporation, Armonk, NY).

## 3. Results

### 3.1. Characteristics of the patients

A total of 245 patients with FIGO stage IB1 to IIA2 cervical cancer who underwent radical hysterectomy type C with pelvic lymphadenectomy were included in the study based on the inclusion and exclusion criteria. MIS was performed in 59 cases (24.1%), and open surgery was performed in 186 cases (75.9%). The baseline clinicopathological characteristics of the patients are summarized in Table 1. There were no significant differences between the groups in terms of age, tumor size, FIGO stage, or histologic subtype. However, patients who underwent MIS had less stromal (*P* < .001) and lymphovascular invasion (23.7% vs 47.8%, *P* = .001) than those who underwent open surgery. The remaining tumor characteristics, such as parametrial invasion (*P* = .413), vaginal margin involvement (*P* = .577), and pelvic lymph node metastasis (*P* = .873), were similar between the groups. Patients who underwent MIS were significantly less likely to require adjuvant therapy (39.0% vs 65.1%, *P* < .001).

**Table 1:** The baseline clinicopathologic characteristics of the patients.

Characteristics	Minimally invasive surgery (N = 59)	Open surgery (N = 186)	*P* value
Age - yr	50.36 ± 11.784	50.09 ± 10.473	.867
Tumor size - centimeters	2.57 ± 1.702	2.74 ± 1.678	.491
FIGO stage of disease - no. (%)			.961
IB1	22 (37.3)	68 (36.6)	
IB2	20 (33.9)	61 (32.8)	
IB3	11 (18.6)	35 (18.8)	
IIA1	2 (3.4)	11 (5.9)	
IIA2	4 (6.8)	11 (5.9)	
Histologic subtype - no. (%)			.630
Squamous cell carcinoma	50 (84.7)	150 (80.6)	
Adenocarcinoma	6 (10.2)	28 (15.1)	
Other types	3 (5.1)	8 (4.3)	
Stromal invasion - no. (%)			<.001
Superficial	40 (67.8)	65 (34.9)	
Middle	11 (18.6)	55 (29.6)	
Deep	8 (13.6)	66 (35.5)	
Lymphovascular invasion - no. (%)	14 (23.7)	89 (47.8)	.001
Parametrial extension - no. (%)	5 (8.5)	23 (12.4)	.413
Vaginal margin involvement - no. (%)	3 (5.1)	16 (8.6)	.577
Pelvic lymph node metastasis - no. (%)	9 (15.3)	30 (16.1)	.873
Adjuvant therapy	23 (39.0)	121 (65.1)	<.001
Chemotherapy only	1 (1.7)	1 (.5)	.424
Radiation only	4 (6.8)	50 (26.9)	.001
Chemoradiation	18 (30.5)	70 (37.6)	.320

FIGO = federation international of gynecology and obstetrics.

### 3.2. Survival outcomes by surgical approach

Among the 245 patients, 24 (9.8%) experienced recurrences, and 12 died (4.9%). In the MIS group, there were 7 (7/59, 11.9%) recurrences and 5 (5/59, 8.5%) deaths. The median follow-up period was 51.75 months. In the open surgery group, there were 17 (17/186, 9.1%) recurrences and 7 (7/186, 3.8%) deaths. The median follow-up period was 76.29 months, which was significantly longer than that of the MIS group (*P* < .001).

**Figure 1.**
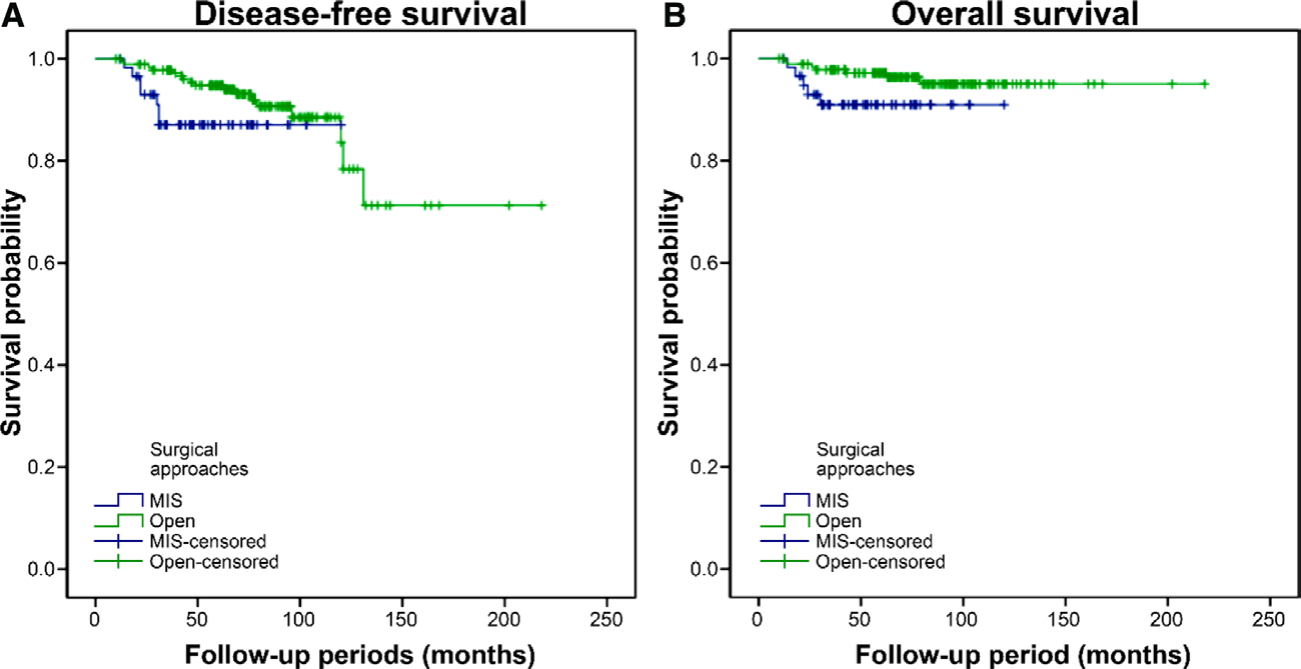
Survival outcomes of minimally invasive surgery and open surgery. (A) Disease-free survival (Log-rank test; *P* value = .088). (B) Overall survival (Log-rank test; *P* value = .072).

Patients who underwent MIS showed similar DFS (*P* = .088) and OS (*P* = .072) to those who underwent open surgery (Fig. 1). There were no significant differences in survival outcomes based on the surgical approach.

### 3.3. Prognostic factors

Table 2 shows the results of univariate and multivariate Cox regression analyses of DFS and OS in all patients. Open surgery was a favorable prognostic factor for DFS (adjusted hazard ratio [HR]: 230; 95% confidence interval [CI]: 086–0.614, *P* = .003) and OS (adjusted HR: 135; 95% CI: 0.41–0.451, *P* = .001). Adjuvant therapy was a poor prognostic factor for DFS (adjusted HR: 6.546; 95% CI: 1.384–30.952; *P* = .018), and deep stromal invasion showed a worsening trend in DFS (adjusted HR: 2.233; 95% CI: 900–5.540; *P* = .083). The deep stromal invasion was identified as an unfavorable prognostic factor for OS (adjusted HR: 8.715; 95% CI: 1.636–46.429; *P* = .011), and lymphovascular invasion tended to be associated with poor OS (adjusted HR: 7.595; 95% CI: 0.832–69.439; *P* = .072).

**Table 2:** Univariate and multivariate Cox regression analyses for disease-free survival and overall survival in all patients.

Risk factors	Disease-free survival				Overall survival			
	Univariate analysis		Multivariate analysis		Univariate analysis		Multivariate analysis	
	Unadjusted HR (95% CI)	*P* value	Adjusted HR (95% CI)*	*P* value	Unadjusted HR (95% CI)	*P* value	Adjusted HR (95% CI)**	*P* value
Age	0.994 (0.956–1.033)	.757	N/A	N/A	0.549 (0.930–1.039)	.549	N/A	N/A
Tumor size	1.319 (1.111–1.566)	.002	N/A	N/A	1.489 (1.177–1.884)	.001	N/A	N/A
Histologic subtype (SCC vs non-SCC)	1.594 (0631–4.028)	.325	N/A	N/A	1.457 (0.394–5.382)	.574	N/A	N/A
Stromal invasion (superficial and middle vs deep)	3.158 (1.401–7.117)	.006	2.233 (0.900–5.540)	.083	11.642 (2.550–53.159)	.002	8.715 (1.636–46.429)	.011
Lymphovascular invasion (absence vs presence)	2.788 (1.184–6.562)	.019	N/A	N/A	14. 661 (1.892–113.591)	.010	7.595 (0.832–69.349)	.072
Parametrial invasion (absence vs presence)	1.343 (0.457–3.943)	.592	N/A	N/A	2.396 (0.646–8.878)	.191	N/A	N/A
Vaginal margin involvement (absence vs presence)	1.835 (0.544–6.186)	.328	N/A	N/A	1.155 (0.149–8.952)	.891	N/A	N/A
Pelvic lymph node metastasis (absence vs presence)	1.766 (0.701–4.451)	.228	N/A	N/A	3.842 (1.219–12.108)	.022	N/A	N/A
Adjuvant therapy (absence vs presence)	7.321 (1.720–31.162)	.007	6.546 (1.384–30.952)	.018	46.995 (0.398–5548.131)	.114	N/A	N/A
Surgical approach (MIS vs open)	0.461 (0.185–1.149)	.096	0.230 (0.086–0.614)	.003	0.361 (0.113–1.149)	.085	0.135 (0.041–0.451)	.001

All variables included in the univariate regression (*P* value < .1 were included in the multivariate regression analysis.

CI = confidence interval, HR = hazard ratio, MIS = minimally invasive surgery, N/A = not available, SCC = squamous cell carcinoma.

* Adjusted for tumor size, stromal invasion, lymphovascular invasion, adjuvant therapy, and surgical approach.

** Adjusted for tumor size, stromal invasion, lymphovascular invasion, pelvic lymph node metastasis, and surgical approach.

## 4. Discussion

We compared the survival outcomes between MIS and open surgery for radical hysterectomy in patients with 2018 FIGO stage IB1 to IIA2 cervical cancer. There were some differences in baseline clinicopathological characteristics between the 2 groups. Deep stromal and lymphovascular invasion, which are intermediate-risk factors, were significantly higher in the open surgery group. This led to a difference in adjuvant therapy administration, especially radiotherapy, between the MIS and open surgery groups (6.8% vs 26.9%, *P* = .001).

This study found no significant differences in DFS and OS based on the surgical approach. However, the multivariate analysis identified MIS as an independent poor prognostic factor for DFS and OS. Kim et al^[7]^ analyzed the survival outcomes of 148 patients with stage IB1 to IIA2 cervical cancer who underwent minimally invasive or open radical hysterectomy. The mean tumor size in all included patients was 1.63 cm. The MIS and open surgery groups had similar DFS (*P* = .267) and OS (*P* = .952). In a subgroup analysis of patients with tumors >2 cm, the MIS group showed poorer DFS (HR: 5.198; 95% CI: 1.339–20.18, *P* = .017) and similar OS (HR: 4.279; 95% CI: 3556–51.50, *P* = .252). Other retrospective studies have also supported the finding that MIS was associated with poor DFS when the tumor was >2 cm.^[8,9]^ Our study showed that MIS was associated with poor survival outcomes regarding both DFS and OS, which is consistent with the results of the LACC trial. The mean tumor size in this study was >2 cm in both groups, without significant differences between the groups. The FIGO IB3 and IIA2 groups, in which the tumor size was >4 cm, accounted for 25.4% and 24.7% of the MIS and open surgery groups, respectively.

In patients with early cervical cancer with 2 or more intermediate-risk factors, adjuvant radiotherapy is recommended according to the GOG92 trial.^[10]^ A randomized trial of pelvic radiotherapy following radical hysterectomy in patients with stage IB cervical cancer indicated longer progression-free survival and a better trend in OS.^[11]^ Quan et al showed that laparoscopic surgery with adjuvant therapy in patients with stage IB1 to IIA cervical cancer had poorer DFS and OS compared with open surgery with adjuvant therapy, whereas there were no significant differences in the DFS and OS of patients without adjuvant therapy regardless of the surgical approach.^[9]^ Kim et al indicated that adjuvant radiotherapy following radical hysterectomy significantly reduced the rate of recurrence (adjusted HR: 241; 95% CI: 082–0.709; *P* = .010) in patients with stage IBI to IIA cervical cancer with intermediate-risk factors, whereas patients who underwent MIS did not show a positive effect of adjuvant radiotherapy.^[12]^ In our study, patients who underwent open surgery significantly received more adjuvant therapies, especially adjuvant radiotherapy. Adjuvant therapy resulted in better survival outcomes in the open surgery group.

In our study, MIS was an independent poor prognostic factor for survival outcomes in patients with stage IB1 to IIA2 cervical cancer. Several studies^[6,9,13]^ have explained the potential reasons for the inferior survival outcomes of minimally invasive radical hysterectomy in patients with early cervical cancer. These include the use of a uterine manipulator, insufflation of CO_2_ gas, intracorporeal colpotomy, and steep Trendelenburg position.

A strength of this study is that a large number of patients were enrolled at a single institution, sharing a relatively unified management process. However, this study has some limitations. First, selection bias was expected given the study retrospective nature. Second, the sample size in the MIS group was smaller than that in the open surgery group. Third, there were few cases of recurrence or death. These may have affected the outcomes of the data.

In conclusion, we found that MIS may be an independent poor prognostic factor for DFS and OS in patients who undergo radical hysterectomy for stage IB1–IIA2 cervical cancer. Overall, the findings of the study can help improve the management of patients with cervical cancer.

## Acknowledgments

The authors thank Dr Chang-Hwan Seong for his advice on statistical analysis. The present study was supported by a research grant from Dong-A University.

## Author contributions

**Conceptualization:** Jung-Woo Park.

**Data curation:** Hwa Yeon Choi, Jung-Woo Park.

**Formal analysis:** Hwa Yeon Choi.

**Funding acquisition:** Hwa Yeon Choi, Jung-Woo Park.

**Investigation:** Hwa Yeon Choi.

**Methodology:** Hwa Yeon Choi, Jung-Woo Park.

**Project administration:** Jung-Woo Park.

**Supervision:** Jung-Woo Park.

**Writing – original draft:** Hwa Yeon Choi.

**Writing – review & editing:** Hwa Yeon Choi.
